# Clip-guided local duodenectomy for safe and minimal local resection of nonampullary duodenal neoplasms

**DOI:** 10.1186/s12893-022-01771-0

**Published:** 2022-08-29

**Authors:** Takeshi Miwa, Suguru Yamada, Kazuto Shibuya, Katsuhisa Hirano, Hideki Takami, Toru Watanabe, Masamichi Hayashi, Isaku Yoshioka, Yasuhiro Kodera, Tsutomu Fujii

**Affiliations:** 1 Department of Surgery and Science, Faculty of Medicine, Academic Assembly, University of Toyama, 2630 Sugitani, Toyama, 930-0194 Japan; 2 Department of Gastroenterological Surgery (Surgery II), Nagoya University Graduate School of Medicine, Nagoya, Japan

**Keywords:** Clip-guided local duodenectomy, Local duodenectomy, Nonampullary duodenal neoplasms, Endoscopic metal clip, Duodenal adenocarcinoma, Duodenal adenoma

## Abstract

**Background:**

Local duodenectomy and primary closure is a simple option for some nonampullary duodenal neoplasms. Minimizing the resection area while ensuring curability is necessary for safe primary duodenal closure. However, it is often difficult to determine the appropriate resection line from the serosal side. We developed clip-guided local duodenectomy to easily determine the resection range and perform local duodenectomy safely, then performed a retrospective observational study to confirm the safety of clip-guided local duodenectomy.

**Methods:**

The procedure is as follows: placing endoscopic metal clips at four points on the margin around the tumor within 3 days before surgery, identifying the tumor extent with the clips under X-ray imaging during surgery, making an incision to the duodenum just outside of the clips visualized by X-ray imaging, full-thickness resection of the duodenum with the clips as guides of tumor demarcation, and transversely closure by Gambee suture. We evaluated clinicopathological data and surgical outcomes of patients who underwent clip-guided local duodenectomy at two surgical centers between January 2010 and May 2020.

**Results:**

Eighteen patients were included. The pathological diagnosis was adenoma (11 cases), adenocarcinoma (6 cases), and GIST (1 case). The mean ± SD tumor size was 18 ± 6 mm, and the tumor was mainly located in the second portion of the duodenum (66%). In all cases, the duodenal defect was closed with primary sutures. The mean operation time and blood loss were 191 min and 79 mL, respectively. The morbidity was 22%, and all complications were Clavien–Dindo grade II. No anastomotic leakage or stenosis was observed. In the 6 adenocarcinoma patients, all were diagnosed with pT1a, and postoperative recurrence was not observed. The 1-year overall and recurrence free survival rate was 100%.

**Conclusions:**

Clip-guided local duodenectomy is a safe and useful surgical option for minimally local resection of nonampullary duodenal neoplasms such as duodenal adenoma, GIST, and early adenocarcinoma.

## Background

Nonampullary duodenal neoplasms (NADNs) are found in 1–5% of patients referred for upper gastrointestinal endoscopy [1, 2]. They include both mucosal and submucosal lesions, including adenomas, adenocarcinomas, neuroendocrine tumors, and gastrointestinal stromal tumors (GIST), and should usually be treated surgically [3, 4]. Surgical or endoscopic removal of duodenal adenoma is also performed according to endoscopic findings because duodenal adenomas have a risk of progression to adenocarcinoma [5].

Although the standard radical surgery for NADNs is pancreaticoduodenectomy (PD), limited resection, such as local resection, pancreas-sparing duodenectomy and segmental duodenectomy, has been reported to be preferable for benign duodenal neoplasms and early duodenal carcinoma, which do not have a risk of regional lymph node metastasis [4, 6–8]. Full-thickness, local resection of the duodenum and hand-sewn closure of the defect is a simple and safe method and may be the most favorable treatment option for NADNs [3, 8, 9]. However, it is often difficult to determine the optimal resection margin for local resection from the serosal side, especially when the tumor is limited to the mucosa. The minimally sufficient resection margin around the tumor is desirable for safe closure of the defect in local resection of the duodenum. If the resection margin is too wide, the defect cannot be closed with primary sutures and requires an ileum patch or other reconstructions. It is necessary for safe resection to determine the optimal incision to the duodenum, which ensures the minimally sufficient resection margin of the tumor. However, to our knowledge, the optimal method for determining this from the serosal side has not been established.

In this study, we developed a new method of local duodenal resection using endoscopic metal clips to detect the tumor margin intraoperatively from the serosal side by palpation and X-ray imaging, allowing the identification of a minimal and sufficient resection margin. To examine the safety of clip-guided local duodenectomy (CGLD) for NADNs, a retrospective observational study was conducted. Here, we describe our surgical technique and report operative outcome of CGLD for NADNs.

## Methods

### Patient characteristics

CGLD was performed at two regional high-volume centers, University of Toyama (Toyama, Japan) and Nagoya University Graduate School of Medicine (Nagoya, Japan), between January 2010 and May 2020. Medical records were reviewed for clinicopathological and perioperative data. Clinicopathological data included age, sex, tumor location, size, and pathological diagnosis. Perioperative data included operation time, blood loss, The American Society of Anesthesiologists physical status (ASA-PS) classification, onset of oral feeding, hospital stay, and postoperative complications. T classifications of duodenal adenocarcinoma were defined based on the Union for International Cancer Control-Tumor Node Metastasis (UICC TNM) classification of malignant tumors of the small intestine [10]. The tumor location is indicated as the first (D1), second (D2), and third (D3) portion of the duodenum. The incidence of postoperative complications was determined according to the Clavien–Dindo classification [11]. The cases of adenocarcinomas were followed up with tumor markers every 3 months, CT scan every 6 months, and duodenal endoscopy every 1 year.

### Indications for CGLD

Indications for CGLD of duodenal neoplasms were defined as follows: (1) NADNs which locate greater than 2 cm from the ampulla of Vater; (2) histological diagnosis of adenoma, superficial adenocarcinoma, or GIST; (3) lesions up to half the circumference of the duodenal wall on endoscopy; (4) tumor location allowing preservation of the ampulla of Vater; and (5) inability to perform endoscopic resection. If the tumor exceeds half the circumference, segmental resection or local resection followed by reconstruction with jejunum should be performed. The tumor locating pancreatic side of duodenum is difficult to treat with local resection, and is indicated with segmental resection or pancreaticoduodenectomy.

### Surgical technique (Fig. 1)

**Fig. 1:**
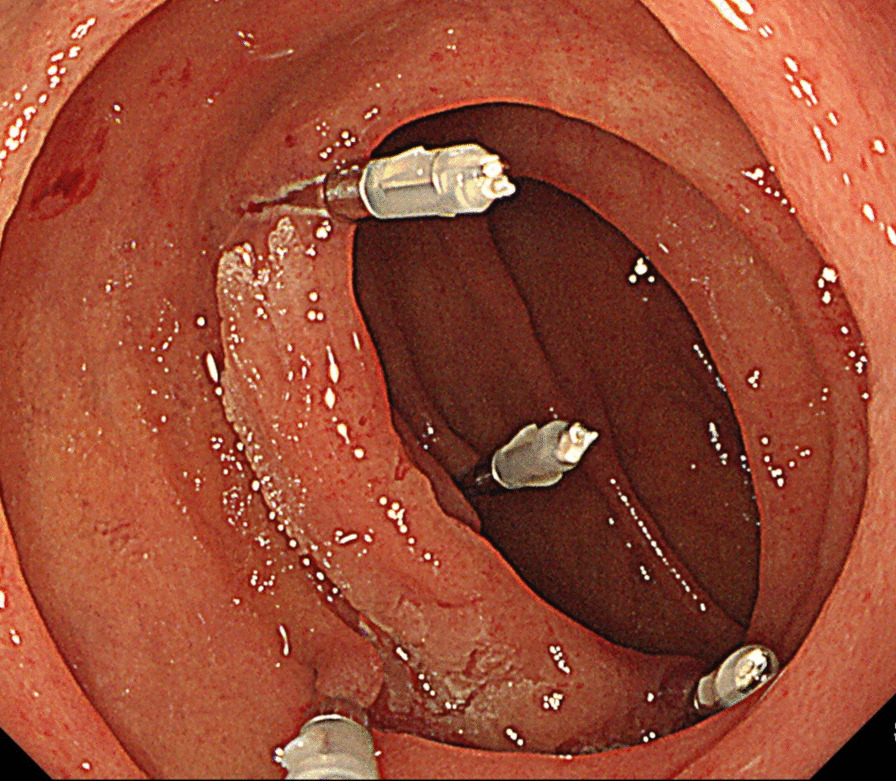
Metal clips were endoscopically placed just along the margin of the tumor 2 or 3 days before surgery

**Fig. 2:**
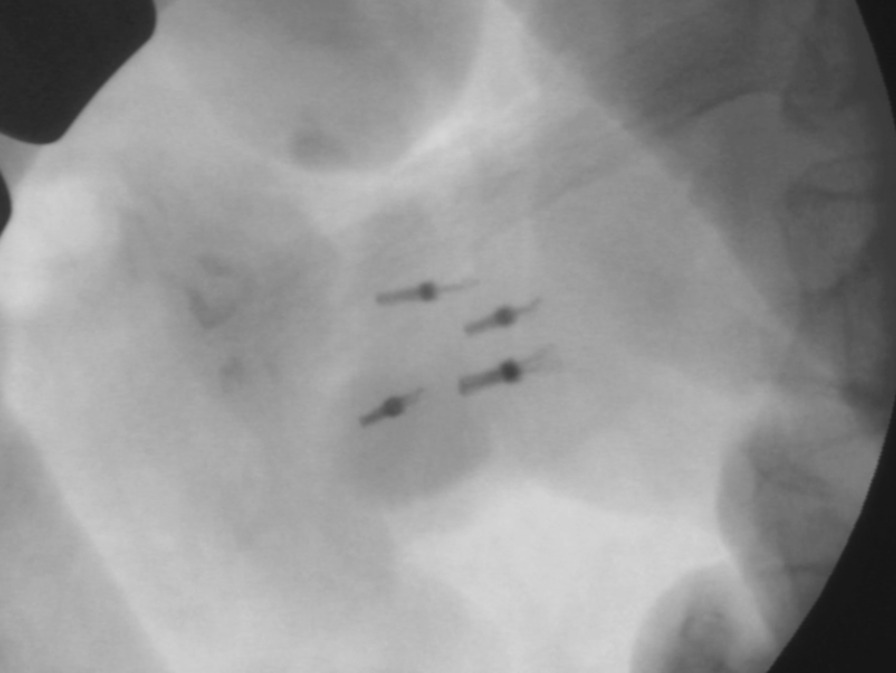
The clips on the margin of the tumor were visible under intraoperative X-ray imaging

**Fig. 3:**
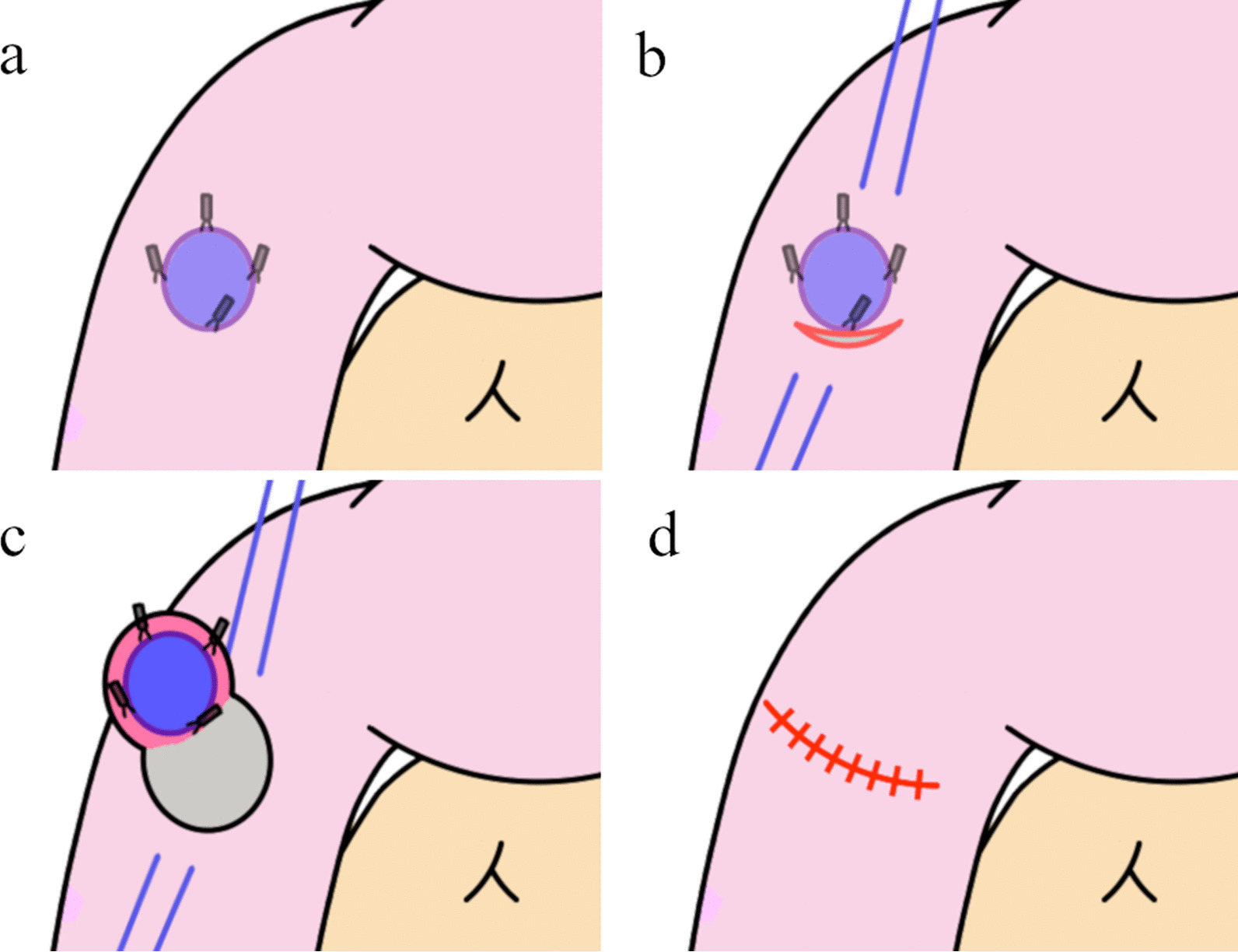
Schematic of the clip-guided local duodenectomy technique. **a** The four clips were detected under X-ray imaging after upper median laparotomy. **b** Stay sutures were placed on both ends of the lesion, and a full-thickness incision in the duodenal wall was performed just outside of the clips under imaging. **c** Full-thickness duodenal resection was performed while directly visualizing the tumor and clips. **d** The defect of the duodenal wall was transversely closed by Gambee sutures

**Fig. 4:**
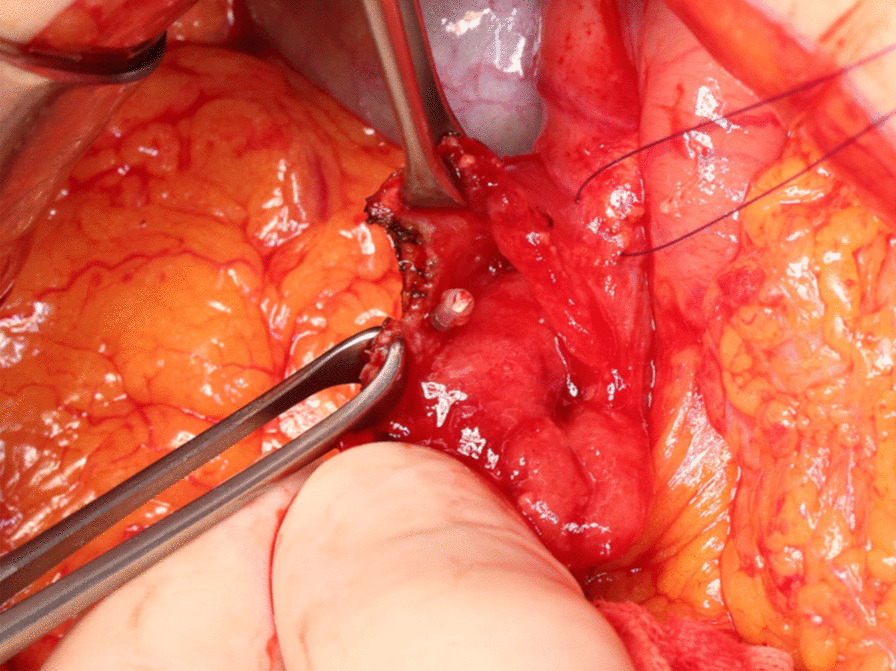
A full-layer incision of the duodenal wall was performed just outside of the clips. The resection of the tumor with minimal margins was achieved by making an incision using the clips as a guide

**Fig. 5:**
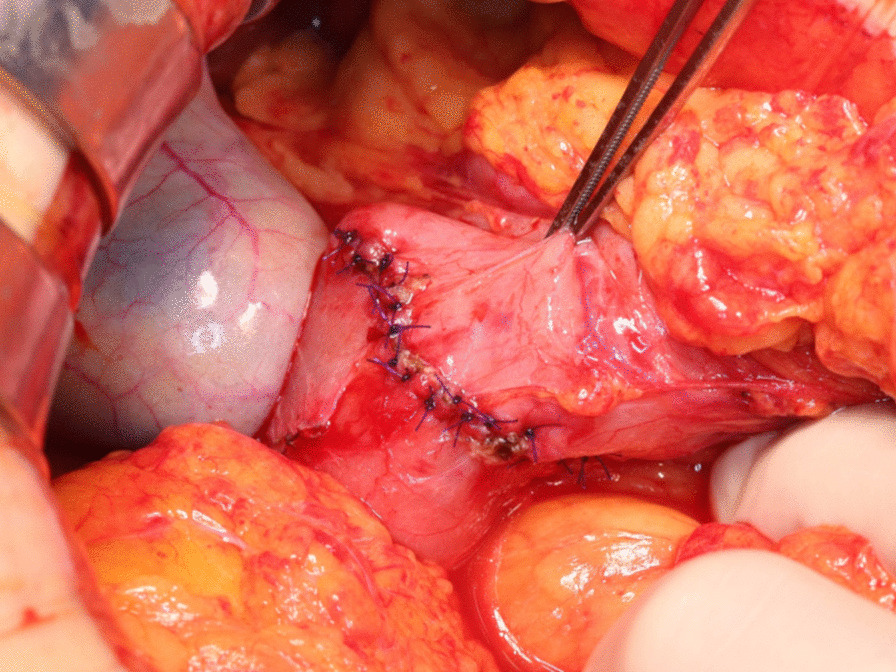
The duodenal defect was finally closed with a single-layer closure by Gambee sutures

All patients preoperatively underwent endoscopy 2 or 3 days before surgery to mark tumors with metal clips. The endoscopists carefully confirmed the margin and placed clips at four points around the tumor (Fig. 1). It is preferable to place the clip before surgery because the intraoperative endoscopy may not be able to accurately locate the tumor margins, and the insufflated gas may cause intestinal dilatation and interfere with the surgical procedure. During surgery, the tumor locations were confirmed by detecting the four clips under X-ray imaging, as well as by palpation, after upper median laparotomy (Figs. 2, 3a). The Kocher maneuver was performed before approaching the tumor, and mobilization of the hepatic flexure and the mesentery of the transverse colon was performed if the tumor was located in D3. Stay sutures were placed on both ends of the lesion, and a full-thickness incision in the duodenal wall was performed just outside of the clips under imaging (Fig. 3b). Tumor excision with full-thickness resection was performed while directly visualizing the tumor and clips (Figs. 3c, 4). The defect of the duodenal wall was transversely closed by Gambee sutures (Figs. 3d, 5). A drainage tube was placed behind the anastomosis for detecting leakage and removed about 7 days after surgery if there were no problems after the patient started oral intake. After fluoroscopy, which was performed usually 4 days after surgery with contrast medium containing iodine, showed that the anastomosis was intact, oral intake was initiated around 5 days after surgery.

## Results

### Clinical characteristics

A total of 23 patients underwent CGLD at 2 regional centers. In all patients, the tumors were easily detected with clip guidance under X-ray imaging, and all duodenal defects were directly closed with hand-sewn sutures. Five patients underwent cooperative surgeries with CGLD, such as hepatectomy and cholecystectomy, were excluded from the analysis. Clinicopathological characteristics and surgical outcomes of the 18 patients are shown in Table 1. There were 13 men and 5 women, aged (mean ± SD) 63.1 ± 11.3 years. The BMI (mean ± SD) was 23.8 ± 2.9 kg/m^2^. Preoperative albumin was 4.1 ± 0.3 g/dL. Two patients had severe systemic diseases (ASA-PS III), namely, chronic renal failure and cerebrovascular disease. Twelve patients had mild systemic disease (ASA-PS II), such as hypertension and diabetes mellitus. The pathological diagnosis was adenoma in 11 patients, adenocarcinoma in 6 patients, and GIST in 1 patient. The tumor depth of all cases of adenocarcinoma was pT1a. The tumor size (mean ± SD) was 17.7 ± 5.8 mm, and the tumor was located in D1 in 1 patient, D2 in 12 patients, and D3 in 5 patients.

**Table 1:** Patient characteristics, surgical outcomes, and postoperative complications

Variables	Data
Background characteristics	
Sex, male:female	13:5
Age, y, mean ± SD	63.1 ± 11.3
BMI, kg/m^2^, mean ± SD	23.8 ± 2.9
ASA-PS, I/II/III	4/12/2
Preoperative albumin, g/dL, mean ± SD	4.1 ± 0.3
Disease, n (%)	
Adenoma	11 (61)
Adenocarcinoma	6 (33)
GIST	1 (6)
Tumor size, mm, mean ± SD	17.7 ± 5.8
Location, n (%)	
D1	1 (6)
D2	12 (66)
D3	5 (28)
Outcomes of surgery	
Operation time, min, mean ± SD	191 ± 72
Blood loss, mL, mean ± SD	79 ± 121
Time to first oral feeding, d, median (range)	7 (2–26)
Hospital stay, d, median (range)	17.5 (9–37)
Negative pathological tumor margin, n (%)	18 (100)
Tumor recurrence, n	0
Mortality, n	0
Postoperative complications	
Morbidity, n (%)	4 (22)
Clavien–Dindo classification, n (%)	
I	0
II	4 (22)
IIIa–V	0
Reoperation, n	0

*ASA-PS* The American Society of Anesthesiologists physical status, *GIST* gastrointestinal stromal tumor, *D1* the first portion of the duodenum, *D2* the second portion of the duodenum, *D3* the third portion of the duodenum

### Operative results and survival

The mean operation time and blood loss were 191 min and 79 mL, respectively. For tumors were located in D1, D2, and D3, the mean operation times were 144, 180, and 226 min, respectively. The operation time tended to be longer when the tumors located in D2 and, moreover, D3. This may be because D2 and D3 lesions require Kocher maneuver and mobilization of transverse mesocolon to approach the tumor. The median days of postoperative fasting and hospital stay were 7 days and 18 days, respectively. No difference was observed in the days of postoperative fasting and hospital stay among patients with different tumor locations, tumor sizes, and pathological diagnoses. The pathological tumor margin was negative in all patients. In the patients with adenocarcinoma, deaths from all causes and recurrences of adenocarcinoma were not observed during the median follow-up period of 883 days (range 197–2090). The 1-year overall and recurrence free survival rate was 100%.

### Complications

The overall postoperative morbidity was 22%. All cases of complications were Clavien–Dindo grade II, and included delayed gastric emptying in 2 (11%) patients, pneumoniae in 1 (6%) patient, and surgical site infection in 1 (6%) patient (Table 2). These complications were resolved by conservative treatment, and no patients had complications of grade III or higher. No patients had anastomotic leakage or other life-threatening complications. Tumor locations and sizes were not associated with postoperative complications. Reoperations and hospital deaths were not observed.

**Table 2:** Details of the postoperative complication

Age	Sex	Location	Complication (C–D grade)	Hospital stay, d
78	Male	D2	DGE (II)	37
61	Male	D2	SSI (II)	29
53	Male	D3	DGE (II)	28
74	Male	D3	Pneumonia (II)	16

*D2* the second portion of the duodenum, *D3* the third portion of the duodenum, *DGE* delayed gastric emptying, *SSI* surgical site infection, *C–D grade* Clavien–Dindo classification grade

## Discussion

The current study evaluated the safety and usefulness of CGLD for NADNs. The clip-guided method helped to achieve minimally sufficient local resection for NADNs. In all patients undergoing CGLD, the defects were safely closed without an ileal patch or anastomotic reconstruction. There was no leakage or stenosis after CGLD. Severe complications and postoperative death were not observed. Additionally, in cases of duodenal pT1a adenocarcinomas, local recurrence and lymph node metastasis did not occur.

A standard surgical strategy for benign duodenal neoplasms and early duodenal carcinoma has not been established [4]. Lymph node metastasis of duodenal carcinoma is a poor prognostic factor [12, 13], and PD is recommended for radical treatment of duodenal carcinoma [14, 15]. These reports were based mainly on cases of T2 or more advanced duodenal carcinoma. Two reports described limited resection for T1 duodenal carcinoma. Kohga et al. [8] reported that none of the 34 patients with T1a duodenal carcinoma developed lymph node metastases, while one of the 5 patients (20%) with T1b disease developed metastasis. Kato et al. [6] reported that lymph node metastasis was not observed in all patients with T1a/T1b duodenal cancer (0/15). Another report mentioned that there were no differences in survival between PD and segmental resection for stage I duodenal cancer [7]. These results indicate that less invasive surgery could be sufficient for curative treatment of T1a duodenal carcinoma. Endoscopic submucosal dissection (ESD) is the standard treatment for early gastric carcinoma and is performed for duodenal adenoma at some institutions [2]. However, as the duodenal wall is thin and the surgical site is exposed to bile and pancreatic juice, duodenal ESD was reported to be associated with immediate and delayed perforation in 39% of patients and delayed bleeding in 18% of patients [16]. Although several methods to endoscopically close the mucosa to prevent perforation have been reported [17], duodenal ESD should be performed by endoscopists who are highly experienced with ESD [18]. Laparoscopic-endoscopic cooperative surgery (LECS) for duodenal tumors could be a favorable and promising treatment option [19, 20], but LECS requires a highly skilled endoscopist to perform endoscopic resection with appropriate margins for duodenal tumors. Thus, the technique and safety of duodenal LECS in general hospitals have not been well established. CGLD does not require an intraoperative endoscopy, and is more feasible when clips could be placed on the tumor margin without such a highly endoscopic technique. Laparoscopic CGLD may be useful as a less invasive approach, especially for D1 and D2 lesions. CGLD, which is an open procedure, can be performed regardless of the technical capabilities of the facility, and it is useful in that it can be performed outside of high-volume centers. Pancreas-sparing duodenectomy and segmental duodenectomy were reported to be useful methods for duodenal limited resection [21, 22]. These methods require reconstruction with intestinal anastomosis or patch closure using the small intestine, possibly with complications, such as leakage and passage disturbance. Additionally, they lead to a wide resection margin of the duodenum if tumors are adenomas or GISTs. Local resection with closure of the defect could result in minimal changes in the duodenum when the resection margin remains in the minimal range, and the defects are able to be safely closed with surgical suturing. It should be adequate to achieve a minimally sufficient margin around the tumor when the tumor does not require resection with lymphadenectomy. However, the optimal incision line for a minimally sufficient resection margin must be determined. We developed a metallic clip-guided technique to solve this problem.

This clip-guided method enables us to easily determine the minimum resection area and to more safely close the defect. In this study, all the tumors could be easily detected under imaging, although almost all the tumors were adenomas or superficial adenocarcinomas, which could not be seen and were unpalpable from the serosal side. In laparoscopic gastrectomy for gastric cancer, the feasibility of marking using dyes such as indocyanine green for location identification has been reported [23]. However, the minimum resection range is pivotal to avoid postoperative stenosis in local duodenal resection, and there is the concern of feathering in marking using dyes resulting in an excessively large resection range. In all cases in this study, defects of CGLD could be closed primarily by hand-sewn sutures, and no additional techniques, such as an ileal patch or anastomotic reconstruction, were required. Additionally, there were no cases of anastomotic leakage or stenosis related to CGLD. Other limited resections, such as segmental duodenectomy and pancreas-sparing duodenectomy, require intestinal reconstruction with anastomosis, which can lead to anastomotic complications and nonphysiological reconstruction. Local resection is the simplest and most well-balanced method among the types of limited duodenal resection. In cases of adenocarcinomas, all were pT1a, and there was no local recurrence, lymph node metastasis, peritoneal metastases or other distant metastasis. When tumors are more advanced, full-thickness resection helps to accurately diagnose the pT factor compared with endoscopic resection. Moreover, CGLD does not require complex reconstruction; thus, it does not disrupt PD when it is found to be necessary. CGLD can be performed without intraoperative endoscopy, although local duodenectomy often requires endoscopy, especially when the tumor is limited to the mucosal side [24]. Furthermore, sometimes the whole tumor cannot be observed by intraoperative endoscopy. In CGLD, the surgeon can easily recognize the clips and entire tumor area. CGLD is performed with opening the lumen of the duodenum, therefore, the possibility of peritoneal dissemination cannot be excluded. We have not experienced any recurrence of peritoneal dissemination. Although careful manipulation is required to prevent leakage of intestinal contents, the risk of dissemination is as well as for other limited resection such as segmental resection and LECS. Clean partial resection should be possible for tumors within 30 mm.

There were some limitations associated with this study. First, this study was a retrospective, single-arm, observational study; thus, CGLD could not be compared with existing surgical techniques. Second, the observational periods were not long; thus, long-term outcomes are unclear. Third, the period of this study was too long; therefore, the length of hospital stay and fasting period were long at the beginning due to historical changes, including those in postoperative management. The anastomosis is exposed to bile and pancreatic juice, and has the high risk of anastomotic leakage, particularly in the case of D2 or D3 lesions. Thus, starting diet tended to be cautious due to concerns about the anastomotic leakage, during the initial phase of the procedure. In recent years, the anastomosis was found to be highly stable, the days of postoperative fasting and hospital stays have tended to shorten. Finally, it is possible that the clips could fall out before surgery. If there are three or fewer clips left next to the tumor, the tumor area could be larger than indicated by the remaining clips. Although we have not experienced any clip fallen out cases, if four clips are not found on intraoperative X-ray imaging, endoscopy should be performed intraoperatively. In addition, this technique requires that clips should be precisely placed just on the margin of the tumor, without leaving any space. If clips are placed away from the tumor, the resected area will be enlarged. Surgeons should discuss clip placement with endoscopists before endoscopy. The clips should be placed at 4 points to ensure adequate tumor area recognition.

## Conclusions

In conclusion, CGLD is a safe and useful method for achieving minimally sufficient local duodenectomy for NADNs. It should be a treatment option for duodenal adenoma, GIST, and early adenocarcinoma.

## Data Availability

The datasets used and analyzed during the current study are available from the corresponding author on reasonable request.

## Abbreviations

NADN: Nonampullary duodenal neoplasm
GIST: Gastrointestinal stromal tumors
PD: Pancreaticoduodenectomy
CGLD: Clip-guided local duodenectomy
ASA-PS: American Society of Anesthesiologists physical status
UICC TNM: The Union for International Cancer Control-Tumor Node Metastasis
D1, D2, D3: The first, second, and third portion of the duodenum
LECS: Laparoscopic-endoscopic cooperative surgery
